# Inoculation against Texas Fever

**Published:** 1899-11

**Authors:** 


### SELECTION.

### INOCULATION AGAINST TEXAS FEVER.

(*Note from the Agricultural Experiment Station of Missouri.*)

For many years Texas fever has been the greatest obstacle in the way of shipping Northern pure-bred cattle to the Southern ranges. Heretofore the losses by this malady in cattle shipped from the North have rarely been less than 40 per cent., and frequently 70 per cent. or more. In the nature of the case, Southern buyers could not pay satisfactory prices and run the risk of loss from Texas fever. To grade up their herds they would willingly buy all of the surplus blooded stock of the North every year at good prices, if by any means the ravages of this fever could be reduced.

For a number of years Dr. J. W. Connaway, of the Missouri Experiment Station, and Dr. M. Francis, of the Texas Experiment Station, with the help of the Missouri Board of Agriculture, have been working on this problem, with the result that a successful treatment has been found and put into operation. Already over 400 blooded cattle have been inoculated and exposed to Texas fever on the ranches for an entire year, with a loss of less than 8 per cent. During this time a loss of 65 per cent. has been reported in one lot sent without inoculation to an adjoining ranch. The climatic conditions and general treatment were similar in the two cases.

*Cause and Nature of Texas Fever.* The cause of Texas fever is a micro-parasite which is found in the blood of Southern-raised cattle. The natural method of communication of this germ is by means of the Texas fever tick (*Boophilus bovis*), which abounds in the South. The disease can also be induced artificially in susceptible cattle by hypodermatic injection of infected blood taken from Southern cattle. When a susceptible animal is infested with ticks or is inoculated with infected blood, the germs thus introduced attack the red blood-corpuscles and destroy them in large numbers. This weakens the vital force of the animal and produces a large amount of broken-down, waste tissue, which must be eliminated from the system. Such a condition demands increased action on the part of the tissues producing the red blood-corpuscles, and larger capacity for carrying off the waste materials through the liver, kidneys, spleen, and bowels. If the animal is able to successfully cope with these exigencies, it will thereafter be immune to the disease and able to resist the action of the fever germ.

*How Immunity may be Attained.* It appears, so far as experiments have yet shown, that the only way of producing immunity is through an actual attack of the disease, in as mild a form as possible, from either infestation with ticks or by inoculation with infected blood. It is thought that Southern-raised cattle become immune while calves by repeated slight attacks of the fever caused by tick-infestation. The Missouri Station first made tests with twenty-one head of young calves by the tick-infestation method, extending over three seasons. Several of the animals that had been well infested with ticks at the North were sent South, and proved to be immune. But on account of the necessity of maintaining quarantined pastures for using these ticks at the North, and the trouble of hand feeding calves from non-immune cows, this method will probably not come into general use. Blood-inoculation tests were begun at the same time, and have thus far proven to be more practical.

*Method of Inoculation with Immune Blood.* The supply animal may be either Southern-raised or one immunized artificially. The blood is taken from the jugular vein of the supply animal through a sterile canula, collected in a clean vessel, and immediately defibrinated by stirring with wire or a glass whipper until the clot is removed. The serum is then transferred to a hypodermatic syringe and injected in proper quantities under the skin at the neck or shoulder of the animals it is desired to protect. All instruments used in this work are thoroughly sterilized and the serum is used while fresh. The dose varies from 1 c.c. to 2.5 or 3 c.c. Severe fever is often produced by a dose of 1 c.c., so the best plan is to give a comparatively small dose at first and repeat if necessary.

The best subjects for inoculation are young cattle from eight to twelve months old, weighing from 500 to 800 pounds. Young calves may also be inoculated while nursing their mothers. But animals recently taken from the cow and not well accustomed to grain diet do not do well. Animals more than twelve months old are much more difficult to immunize, and it is certain that aged bulls and cows cannot be immunized as successfully as young stock.

The most suitable time is at seasons when the animal will not suffer from either extreme heat or cold, although this work has been done at all seasons of the year with success. If inoculated in winter, the cattle must be well sheltered. Cattle should be sent South in December or January, to prevent sudden gross infestation with the ticks there.

*Fever Resulting from Inoculation.* It must always be kept in mind that the inoculation fever is genuine Texas fever, and in some cases death will ensue. Some animals require very careful attention and nursing through the fever resulting from inoculation, and some medical treatment. Unless a fever more or less severe is produced by the inoculation the animal will probably not become immune. In some cases as many as three inoculations were made. After inoculation there is an incubation period of seven or eight days; then the inoculation fever begins and continues for eight or nine days, although in some cases it may not exceed four days, and may be prolonged fifteen days. This is called the primary fever period, and the temperature of the sick animals will usually range from 104° to 106°. Maximum temperatures of 107° to 108° have been noted in a few cases. At the same time the fever rises, the percentage of red corpuscles in the blood begins to decrease, falling from the normal (about 35 per cent. or 40 per cent.) to 20 per cent., and in severe cases as low as 10 per cent. or 15 per cent. At the close of the primary fever period, about the fifteenth day after inoculation, the temperature falls rapidly, and in severe cases the animal may die from collapse. From the fifteenth to the twenty-ninth or thirtieth days the animal will gain in strength, and the percentage of red corpuscles in the blood will return almost to the normal. About the thirtieth day after inoculation a secondary fever period often occurs, lasting seven or eight days. This period is usually not so severe as the primary, but the temperature often shows high elevation, and the destruction of red blood-corpuscles occurs again. At the end of fifty or sixty days the inoculated animals are ready for shipment.

*Care of Cattle during Inoculation.* It is necessary that inoculated animals be well nourished during the fever and subsequently, since there is great lowering of the vitality of the animal, due to the destructive action of the micro-parasites on the blood-corpuscles. In these experiments the food has consisted of ground-oats, bran, crushed corn, linseed-meal, clover, and timothy hay. The effort is made to feed in a manner that will maintain a lax condition of the bowels, since the elimination of the waste products from the liver is mainly through the bowels. It is sometimes necessary to give salts to induce proper action of the bowels.

Other medicines, such as stimulants and tonics, are used as indicated. The animals are kept warm and comfortable, in clean quarters, and in every way carefully nursed. They have grass at the proper season. After sending inoculated animals south, they are watched during the first season to prevent relapse, and care is taken to avoid all conditions that lessen the vitality of the animal, as over-heating, undue excitement, or too much service.

*Further Experiments Now in Progress.* The Missouri Experiment Station is now inoculating at Columbia about 250 head of cattle, representing the Shorthorn, Hereford, Devon, and Red Polled breeds. These will be exposed to the fever in the South next summer for a further test of the efficiency of this method of immunizing cattle against this disease. The success of this method has already had the effect of greatly increasing the number of blooded cattle bought in the North by Texas cattlemen, and will add much to the value of all blooded breeding cattle in the North. Through this means a large and important market, which has heretofore been practically closed, is now opened to the Northern breeder. An illustrated *Bulletin*, No. 48, giving full particulars of these experiments, is now being issued by the Missouri Station, and will be sent free to all parties interested upon application.

---

The loss of six cows of Miller & Sibley, of Franklin, Pa., from the forcing of additional milk into the udders while on exhibition at the "Toronto Fair Exposition" has brought forth a manly letter from the firm, completely denying any responsibility for the same or knowledge of their employé, who, in his thoughtless zeal to win honors, was utterly forgetful of the fatal possibilities thereof, not to think of the great dishonor and reflection upon the firm. Messrs. Miller & Sibley have very honorably declined to accept any of the medals or prizes awarded them under these distressing circumstances.

"Breeds of Dairy Cattle," by Henry E. Alvord, Chief of the Dairy Division of the Department of Agriculture, is an excellent compilation and review of the merits, producing qualities, and value of the various breeds of cattle for dairy purposes. The tables of the products of the various breeds and the points for judging decided upon by the various cattle associations add much to the value of the book, and it should be in the hands of every veterinarian to better acquaint him with this branch of animal industry.

